# *Lycium intricatum* Boiss.: An unexploited and rich source of unsaturated fatty acids, 4-desmethylsterols and other valuable phytochemicals

**DOI:** 10.1186/s12944-015-0055-9

**Published:** 2015-06-24

**Authors:** Abdennacer Boulila, Afef Bejaoui

**Affiliations:** Laboratory of Natural Substances, National Institute of Research and Physico-chemical Analysis, Biotechpole of Sidi Thabet, Ariana, 2020 Tunisia; Laboratory of Plant Biotechnology, National Institute of Applied Sciences and Technology, BP 676, Centre Urbain Nord, Tunis Cedex, 1080 Tunis Tunisia

**Keywords:** *Lycium intricatum*, Seed oil, Fatty acids, Phytosterols, Vitamin D

## Abstract

**Background:**

*Lycium intricatum* Boiss., a Solanaceous shrubbery is used in Tunisia as a windbreak and medicinal plant. However, it is considered as underexploited specie despite its high potential to serve as source with economic and nutritional value. To date only limited information about its phytochemistry, especially of its oil has been published. This work provides data on fatty acids, phytosterols and vitamin D composition of *L. intricatum* seed oil. It opens up new possibilities of developing *L. intricatum* as a new crop that contains phytochemicals with high added value little influenced by selection or commercial breeding.

**Findings:**

The composition of fatty acids, phytosterols and vitamin D in *L. intricatum* seed oil was assessed by GC-FID.

The main fatty acids of *L. intricatum* seed oil were linoleic acid (49.47 %), palmitoleic acid (27.96 %) and erucic acid (13.62 %). Palimtic acid was present at low percentage (0.63 %). The content of unsaturated fatty acids was high as 94.04 %. The sterolic fraction was composed of stigmasterol (18.56 mg/100 g), β-sitosterol (13.04 mg/100 g). *L. intricatum* oil is an oily matrix that contains hydrocarbons, mainly squalene (63.36 mg/100 g), and two triterpenic alcohol erythrodiol (80.36 mg/100 g) and uvaol (24.06 mg/100 g). provitamin D was present in high quantity (8.12 mg/100 g).

**Conclusions:**

From these results it has been shown that *L. intricatum* seeds have great potential as a source of fatty acids and phytosterols for natural health products.

## Additional non-English language abstract

La composition en acides gras et en phytosterols des graines de *Lycium intricatum* Boiss. (Famille des Solanacées), de haute valeur nutritionnelle, a été étudiée moyennant la chromatographie en phase gazeuse (GC-FID). L’huile des graines de *L. intricatum* est caractérisée par des fortes teneurs en acide linoléique (49,47 %), acide palmitoleique (27,96 %) et acide erucique (13,62 %). Les acides gras insaturés présentent 94,04 % de la composition totale en acides gras. La fraction stérolique est composée du stigmasterol (18,56 mg/100 g) et du ß-sitosterol (13,04 mg/100 g). L’huile de *L. intricatum* est principalement composée par des hydrocarbures tels que le squalène (63,36 mg/100 g) et deux alcool triterpeniques qui sont l’erythrodiol (80,36 mg/100 g) et l’uvaol (24,06 mg/100 g). La provitamine D présente une teneure de 8,12 mg/100 g.

## Background

The fatty acid composition of oils from vegetable sources varies depending on plant origin, genetic factors, ripening grade of fruits and specific climatic conditions. In addition to fatty acids, vegetable oils contain phytosterols which are divided into three main classes: 4-desmethylsterols (sterols), 4-monomethylsterols and the 4,40-dimethylsterols [1].

The unsaponifiable fraction of vegetable oils contains a variety of bioactive substances, which include sterols, hydrocarbons, tocopherols, terpenes and others. These minor compounds are more characteristic of each fat and oil [2, 3]. Moreover, it has been reported that phytosterols, which represent the predominant portion of unsaponifiable matter, have multifunctional properties, including anti-inflammatory, antitumor, hypercholesterolemia, antifungal and antibacterial activities [4–6].

The genus *Lycium* (Solanaceae family) has been identified as a rich source of polysaccharidic, proteins and particularly glycopeptides, which are responsible for many health related benefits of this plant. *Lycium* sp. contains 18 different amino-acids, including eight essential amino-acids. The genus includes more than 70 species growing in temperate to subtropical parts of North and South America, Southern Africa, Eurasia, and Australia [7]. *Lycium* sp. is well known as a traditional herbal medicine and functional food. Among the chemical constituents of *Lycium* fruits, the most well researched components are anthocyanins and flavonoids [8]. Recent studies indicate that extracts from some *Lycium* species possess a range of biological activities, including effects on ageing, neuroprotection, anti-fatigue/endurance, glucose control in diabetics, and antioxidant and anti-tumour properties [9, 10].

In Tunisia, four *Lycium* species have been identified: *Lycium europaeum* L., *L. halimifolium* Mill., *L. arabicum* Boiss., and *L. intricatum* Boiss. [11]. *L. intricatum* Boiss. is a common fleshy-fruited, thorny shrub up to 3 min height, typical of sub-humid and semi arid bioclimatic zones in Tunisia. It produces berries that are red when ripe. It is used as a hedge and as wind break plant. In addition the dry powder of its fruit was used to protect from eye diseases. However, in Tunisia *L. intricatum* is considered as underexploited specie despite its high potential to serve as source with economic and nutritional value. An improved knowledge about its chemical composition and biological activities would contribute to the use of this natural resource as a source of phytochemicals as well as to agronomic and economic advancement.

To the best of our knowledge, although the potential beneficial effects of *L. intricatum* were obvious, there are no report in the literature concerning its fatty acids and phytosterols composition. The aim of this study was to determine for the first time the fatty acid composition and phytosterol content of *L. intricatum* seed oil.

## Results and discussion

### Fatty acid profiles by GC

**Fig. 1:**
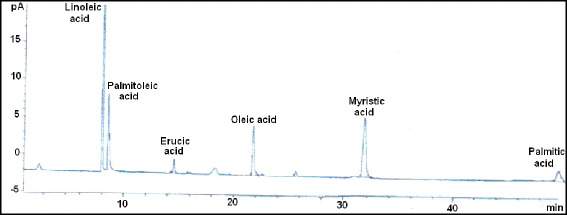
Gas chromatograph of *L. intricatum* fatty acids. pA: Picoampere; min: minute

The yield of seed oil of *L. intricatum* aerial parts was 20 % (±3). Seed oil yield (w/w) as calculated on the basis of dry matter weight. A total of six different fatty acids were identified (Table 1 and Fig. 1). In *L. intricatum* oil, linoleic acid was the dominating fatty acid with an exceptional level, up to 49.47 % followed by palmitoleic acid (27.96 %) and erucic acid (13.62 %). Linoleic acid is an essential fatty acid and a precursor of arachidonic acid biosynthesis, the substrate for eicosanoid synthesis. According to Keys et al. [12], linoleic acid has hypocholesterolemia effects.

**Table 1:** Fatty acid composition (in %) of *L. intricatum* seeds

Fatty acid		Percentage (%)
Myristic acid	C14:0	5.3
Palmitic acid	C16:0	0.63
Palmitoleic acid	C16:1	27.96
Oleic acid	C18:1	2.99
Linoleic acid	C18:2	49.47
Erucic acid	C22:1	13.62
Saturated fatty acid	5.93	
Monounsaturated fatty acid	44.57	
Polyunsaturated fatty acid	49.47

The content of unsaturated fatty acids was 94.04 %. Our results are not in agreement with those published by Altintas [13] for *L. barbarum* oil: hexadecanoic acid (47.5 %), linoleic acid (9.1 %), myristic acid (4.2 %) and ethylhexadecanoate (4 %). However, saturated fatty acids (SFA) fraction was characterized by a lower level (5.93 %). Recently, it was proven by clinical evidence that PUFAs are able to alleviate symptoms of certain diseases such as coronary heart disease, stroke and rheumatoid arthritis [14]. Also, linoleic acid has beneficial properties for skin, and for this purpose it is used by the cosmetics products industry [15]. These results bring attention to the possible use of cactus seed oil as a natural source of PUFAs for nutritional, industrial or pharmaceutical purpose. Indeed, different means are used to increase, directly or indirectly, the human consumption of PUFAs [16].

Oleic acid and linoleic acid exhibited contrasting accumulation patterns in *L. intricatum* fruit. The percentage of linoleic acid was 49.47 % whilst the oleic acid percentage 2.99 %. This is probably the consequence of an alteration of the desaturation step from oleic acid to linolenic acid, which is mediated by specific oleate desaturase enzymes. This is in agreement with the results obtained by Guoliang [17]. However, *L. intricatum* and *L. barbarum* present a disadvantage to fast oxidation due to their richness in polyunsaturated fatty acids. On the other hand, the higher content of unsaturated fatty acids (94.04 % *L. intricatum* and 86.5 % *L. barbarum*) which allow them to act as antiatherogenic and hypocholesterolemia agents in these two oils which are a good sources of omega 6 with antiallergic and anti-inflammatory properties.

### Phytosterol analysis by GC

#### Phytosterol (4-desmethylsterol) content

The sterolic fraction was composed by stigmasterol (18.56 mg/100 g), β-sitosterol (13.04 mg/100 g), and ergosterol (8.12 mg/100 g). *L. intricatum* oil contained a higher amount of sterols 39.72 mg/100 g. Similar values were published by Potterat [10] for the sterol content in seed oil of two Goji species (*L. barbarum* and *L. chinense*). Recently, sterols have been added to vegetable oils as an example of a successful functional food [18]. This type of product is now available and has been scientifically proven to lower blood LDL Cholesterol by around 10-15 % as part of a healthy diet [19].

In oil, Provitamin D was represented only by ergosterol. The Vitamin D level was higher in *L. intricatum* oil 8.12 mg/100 g. The high level of Vitamin D, detected in the oil, may contribute to great stability toward oxidation. Ergosterol is the end product of the sterol biosynthetic pathway and is the major sterol in yeasts. Like cholesterol in mammalian cells, it is responsible for membrane fluidity and permeability [20]. Previous works have been reported a role for ergosterol in physiological functions, such as membrane permeability, resistance to drugs, protein transport to the plasma membrane, sporulation and endocytosis [21–23].

**Fig. 2:**
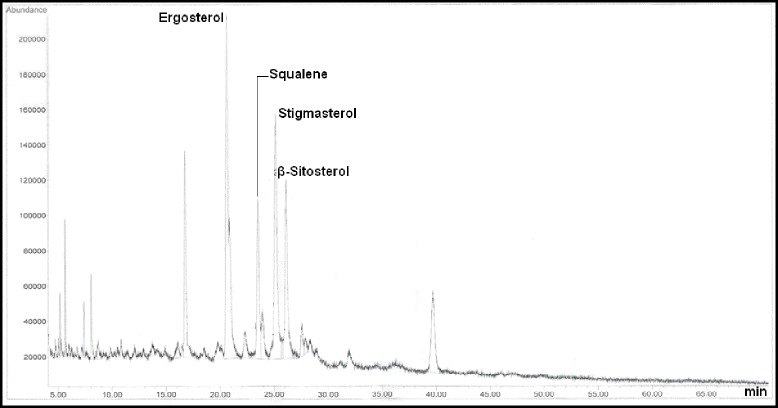
Gas chromatograph of *L. intricatum* sterols. min: minute

A relatively high content of triterpenes such as erythrodiol (80.36 mg/100 g), uvaol (24.06 mg/100 g) and hydrocarbons, mainly squalene (63 mg/100 g) were detected in the seed oils of *L. intricatum* (Table 2 and Fig. 2). Das [24] reported that squalene, a hydrocarbon of lipid composition, exerted antioxidant effects used as food supplement and a vaccine additive.

**Table 2:** Sterols (4-desmethylsterols) composition (mg/100 g of oil) of *L. intricatum* seed

	Phytosterols (mg/100 g)
Provitamine D (Ergosterol)	8,12
Stigmasterol	18.56
β-Sitosterol	13.04
**Total**	**39.72**
Squalene (mg/100 g)	63.36
Triterpenic alcohol di-hydroxyl	
Erythrodiol	80.36
Uvaol	24.06

A similar trend was also observed with hydrocarbons, mainly squalene; triterpenes such as uvaol, erythrodiol in Virgin olive oil [25]. Vuorio et al. [26] and Rajaratnam et al. [27] have been found elevated ratios of serum squalene to cholesterol, leading them to propose that reduced cholesterol synthesis may be related to coronary atherosclerosis [28]. In fact, squalene administration modulates lesion development in a sex-specific manner and that it could be used as a safe alternative to correct hepatic steatosis and atherosclerosis, particularly in males [29].

Uvaol and erythrodiol exhibited antioxidant properties against lipid peroxidation in vitro, and also reduced the generation of hydrogen peroxide by stimulated macrophages in a dose-dependent manner [30–32]. Allouche et al. have shown that uvaol and erythrodiol significantly decreased thrombin formation [30], and they also inhibit cell proliferation in a dose- and time-dependent manner [32]. These compounds have interesting therapeutic potential as cardiovascular drugs.

*L. intricatum* oil is an oily matrix that contains hydrocarbons, mainly squalene; triterpenes such as uvaol, and erythrodiol, phytosterols, and a wide range of phenolic compounds comprising polyphenols, flavonoids, and anthocyanins. The 4-desmethylsterols are the final products of the phytosterols biosynthesis; this category corresponds to plant sterols accumulated mainly in the fruit and seeds. These compounds display anti-inflammatory properties and have long been considered to be the main active principle of *Lycium* sp [33].

## Conclusions

This study shows that *Lycium intricatum* seed oil was found to possess 94.04 % PUFA. Linoleic acid was the dominating fatty acid with an exceptional level, up to 49 %. Oil contained a higher amount of sterols. These results bring attention to the possible use of *Lycium* seed oil as a natural source of PUFA for nutritional, industrial or pharmaceutical purpose. *L. intricatum* oil is an oily matrix that contains hydrocarbons, mainly squalene, and triterpenes such as uvaol and erythrodiol. This study helps to develop *L. intricatum* as a new crop for oil production.

## Methods

### Chemicals

Solvents of analytical (n-hexane, petroleum ether, chloroform, EtOH and MeOH) grade were purchased from Carlo Erba Reactif-CDS (Val de Reuil, France). Sodium sulfate (Na_2_SO_4_), sodium methylate (CH_3_ONa), potassium hydroxide (KOH), chlorhydric acid (HCl) were obtained from Merck (Darmstadt, Germany). The standards α-cholestanol, and 3,3-bis(4-hydroxyphényl)-1-(3H)-monobenzofuranone (φφ) were sourced from Sigma–Aldrich (St. Louis, MO).

### Plant material

While there are no signs of toxicity of this plant, Tunisian peoples collect its fruits only in the full maturity stage to avoid risk of toxicity. So, fruits of *L. intricatum* were collected in May 2013, from the region of Sidi Thabet, area of Ariana (Northern Tunisia, latitude 36°54’45.25”N, longitude 10°06’02.10”E, altitude 30 m) at full maturity stage. Seeds (3 × 25 g) were carefully separated from fruits prior to extraction.

### Lipid extraction

Oil was extracted by a soxhlet extractor for 6 h using n-hexane, chloroform and petroleum ether as solvent. The solvent was evaporated under reduced pressure, using a rotary evaporator at 40 °C. The oil content was determined as the difference in weight of dried *L. intricatum* seed sample before and after the extraction [34]. Oil was weighed and stored at −20 °C. All the analyses were conducted in triplicate.

### Fatty acid methyl ester (FAME) preparation and gas chromatography analysis

In 25 mL round bottom flask, oil samples (3 g) were added to 3 mL methylate sodium solution with φφ. Mixture was refluxed for 10 min, and 3 mL of MeOH solution of hydrochloric acid (a mixture of hydrochloric acid gas and anhydrous methanol or, acetyl chloride and anhydrous methanol) were added to φφ discoloration. The mixture was refluxed for 10 min and then cooled at room temperature.

Hexane (8 mL) and water (10 mL) were added and the organic phase was recovered, dried over anhydrous sodium sulphate and filtered for subsequent GC analysis. FAMEs were analyzed by gas chromatography (GC) using a Agilent 6890 chromatograph series using a Innowax capillary column with the following characteristics: length, 50 m; internal diameter, 0.30 mm; film thickness 0.25 μM. The carrier gas was helium, at a flow through the column of 1 mL/min. The injector temperature was maintained at 230 °C and the flame-ionisation detector (FID) at 250 °C. The oven temperature was 165 °C. The fatty acids were identified by comparison of their retention times with those of standards (COI/T.20/Doc. n° 24).

### Saponification

Unsaponifiable lipids were determined by saponifying 5 g of lipid extracts with 50 mL ethanolic KOH 2 N (w/v) mixed with both 0.5 mL α-cholestanol solution (internal standard: 0.2 % (w/v)) and heating at 60 °C for 1.30 h. After cooling, 50 mL of H_2_O was added and the unsaponifiable matter was extracted four times with 80 mL ethylic ether. The combined ether extract was washed with 50 mL of H_2_O. The ether extracted was dried over anhydrous Na_2_SO4 and evaporated. The dry residues were dissolved in chloroform for TLC analysis.

### Thin-layer chromatography

The unsaponifiable matter was separated into subfractions on preparative silica gel thin-layer plates (silica gel 60 G F254), using 1-dimensional TLC with hexane-Et_2_O (65:35 V/V) as the developing solvent. The unsaponifiable (0.3 mL CHCl_3_) containing 1 % (w/w) of α-cholestanol as the internal standard for 4-desmethylsterols was applied on the silica gel plates in 3 cm bands. To correctly identify the sterols bands, a reference sample of purified sterol (α-cholestanol) were applied on the left side of the TLC plates. After development the plate was sprayed with 2’,7’-dichlorofluorescein and viewed under UV light. On the basis of the reference spot, the sterols band was identified. The band corresponding to 4-desmethylsterols was scraped off separately and was extracted three times with CHCl_3_-Et_2_O (1:1), filtered to remove the residual silica, dried in a rotary evaporator and stored at 10 °C for further analysis.

### GC analysis

Each sterol fraction was silylated with 100 μL of Bis (trimethylsilyl) trifluoroacetamide + 1 % trimethylchlorosilane agent at 6 °C for 30 min. Phytosterols content was determined using gas chromatography. An Agilent 6890 N capillary gas chromatograph equipped with an automatic split/splitless injection (1 μL), flame-ionization detector and HP-5 MS column (30 m × 0.25 mm I.D., 0.25 μm film thickness) was used. Operating conditions were: injection temperature, 260 °C; detector temperature, 320 °C, oven temperature 140 °C to 300 °C, at 10 °C/min, holding at 300 °C for 14 min, helium as carrier gas (1.9 mL/min). Identification and quantfication were based on external standards using ergosterol, stigmasterol and β-sitosterol from Sigma-Aldrich Inc (St. Louis, MO) [35].

## References

[1] Velasco L, Rojas-Barros P, Fernandez-Martinez JM (2005). Fatty acid and tocopherol accumulation in the seeds of a high oleic acid castor mutant. Ind Crop Prod.

[2] Alain R, Maryse T, Pierrette B-N, Paulette S, Pierre B, Francis S, Acharan SN, Luigi C, Claude A, Pierre P (1986). Design of high energy intermediate analogues to study sterol biosynthesis in higher plants. Lipids.

[3] Akihisa T, Inada Y, Ghosh P, Thakur S, Rosenstem FU, Tamura T, Matsumoto T (1988). Composition of triterpene alcohols of seeds and mature plants of the family, Cucurbitaceae. JAOCS.

[4] Antonio LL, Alfredo M, Maria Victoria RM, Antonio GF (2008). Sterols, fatty alcohols, and triterpenic alcohols. Commercial Table Olives.

[5] Harwood J, Aparicio R, Analysis and Properties (1999). Handbook of Olive Oil. Analysis and Properties (Ed.).

[6] Aparicio R, Mcintyre P, Lees M (1998). Oils and fats. Food Authenticity: Issues and Methodologies.

[7] Fukuda T, Yokoyama J, Ohashi H (2001). Phylogeny and biogeography of the genus *Lycium* (Solanaceae): Inferences from chloroplast DNA sequences. Mol Phylogenet Evol.

[8] Boulila A, Mattoussi K, Mrabet Y, Rokbeni N, Dhouioui M, Boussaid M (2015). Determination of phytochemicals and antioxidant activity of methanol extracts obtained from the fruit and leaves of Tunisian *Lycium intricatum* Boiss. Food Chem.

[9] Chen Z, Tan BK, Chan SH (2008). Activation of T lymphocytes by polysaccharide–protein complex from *Lycium barbarum* L. Int Immunopharm.

[10] Potterat O (2010). Goji (*Lycium barbarum* and *L. chinense*): Phytochemistry, pharmacology and safety in the perspective of traditional uses and recent popularity. Planta Med.

[11] Pottier-Alapetite G (1981). Flore de la Tunisie Angiospermes-Dicotylédones, Apétales Dialypétales. Imprimerie Officielle de la République Tunisienne.

[12] Keys A, Anderson JT, Grande F (1957). Prediction of serum cholesterol response of man to change in fats in the diet. Lancet.

[13] Altintas A, Kosar M, Kirimer N, Baser KHC, Demirci B (2006). Composition of the essential oils of *Lycium barbarum* and *L. ruthenicum* fruits. Chem Nat Compd.

[14] Calder PC (2008). Polyunsaturated fatty acids, inflammatory processes and inflammatory bowel diseases. Mol Nutr Food Res.

[15] Darmstadt GL, Mao QM, Chi E, Saha SK, Ziboh VA, Black RE, Santosham M, Elias PM (2002). Impact of tropical oils on the skin barrier: possible implications for neonatal health in developing countries. Acta Paed.

[16] Lewis TE, Nichols PD, McMeekin TA (1999). The biotechnological potential of thraustochytrids. Marine Biotechnol.

[17] Guoliang L, Junyou S, Yourui S, Zhiwei S, Lian X, Jie Z, Jinmao Y, Yongjun L (2011). Supercritical CO_2_ cell breaking extraction of *Lycium barbarum* seed oil and determination of its chemical composition by HPLC/APCI/MS and antioxidant activity. Food Sci Technol.

[18] Ntanios F (2001). Plant sterol-ester-enriched spreads as an example of a new functional food. Eur J Lipid Sci Technol.

[19] Jones P, Raeini-Sarjaz M, Ntanios F, Vanstone C, Feng J, Parsons W (2000). Modulation of plasma lipid levels and cholesterol kinetics by phytosterol versus phytostanol esters. J Lipid Res.

[20] Parks LW, Smith SJ, Crowley JH (1995). Biochemical and physiological effects of sterol alterations in yeast – a review. Lipids.

[21] Proszynski TJ, Klemm RW, Gravert M, Hsu PP, Gloor Y, Wagner J (2005). A genome-wide visual screen reveals a role for sphingolipids and ergosterol in cell surface delivery in yeast. Proc Natl Acad Sci U S A.

[22] Enyenihi AH, Saunders WS (2003). Large-scale functional genomic analysis of sporulation and meiosis in Saccharomyces cerevisiae. Genetics.

[23] Kishimoto T, Yamamoto T, Tanaka K (2005). Defects in structural integrity of ergosterol and the Cdc50p-Drsp putative phospholipids translocase cause accumulation of endocytic membranes, onto which actin patches are assembled in yeast. Mol Biol Cell.

[24] Das B (2000). The Science Behind Squalene. In *The Human Antioxidant* (Ed.).

[25] Josè M, Lou-Bonafonte CA, Maria A, Navarro JO (2012). Efficacy of bioactive compounds from extra virgin olive oil to modulate atherosclerosis development. Mol Nutr Food Res.

[26] Vuorio AF, Miettinen TA, Turtola H, Oksanen H (2002). Cholesterol metabolism in normal and heterozygous familial hypercholesterolemic newborns. J Lab Clin Med.

[27] Rajaratnam RA, Gylling H, Miettinen TA (1999). Serum squalene in postmenopausal women without and with coronary artery disease. Atherosclerosis.

[28] Rajaratnam RA, Gylling H, Miettinen TA (2000). Independent association of serum squalene and noncholesterol sterols with coronary artery disease in postmenopausal women. J Am Coll Cardiol.

[29] Guillen N, Acin S, Navarro MA, Perona JS (2008). Squalene in a sex-dependent manner modulates atherosclerotic lesion which correlates with hepatic fat content in apoE-knockout male mice. Atherosclerosis.

[30] Allouche Y, Beltran G, Gaforio JJ, Uceda M (2010). Antioxidant and antiatherogenic activities of pentacyclic triterpenic diols and acids. Food Chem Toxicol.

[31] Marquez Martin A, de la Puerta VR, FernandezArche A, Ruiz-Gutierrez V (2006). Supressive effect of maslinic acid from pomace olive oil on oxidative stress and cytokine production in stimulated murine macrophages. Free Radic Res.

[32] Allouche Y, Warleta F, Campos M, Sanchez-Quesada C (2011). Antioxidant, antiproliferative, and pro-apoptotic capacities of pentacyclic triterpenes found in the skin of olives on MCF-7 human breast cancer cells and their effects on DNA damage. J Agric Food Chem.

[33] Dell’Agli M, Di Lorenzo C, Badea M, Sangiovanni E, Dima L, Bosisio E (2013). Plant food supplements with anti-inflammatory properties: A systematic review (I). Crit Rev Food Sci Nutr.

[34] AOCS (1989) Official methods and recommended practices of the American oil chemist’s Society. Champaign: American Oil Chemist’s Society. Method Ce-66.

[35] Pennisi Forell SC, Ranalli N, Zaritzky NE, Andrés SC, Califano AN (2010). a Effect of type of emulsifiers and antioxidants on oxidative stability, colour and fatty acid profile of low-fat beef burgers enriched with unsaturated fatty acids and phytosterols. Meat Sci.

